# Synapse-Neuron-Aware Training Scheme of Defect-Tolerant Neural Networks with Defective Memristor Crossbars

**DOI:** 10.3390/mi13020273

**Published:** 2022-02-08

**Authors:** Jiyong An, Seokjin Oh, Tien Van Nguyen, Kyeong-Sik Min

**Affiliations:** School of Electrical Engineering, Kookmin University, Seoul 02707, Korea; sunday1903@kookmin.ac.kr (J.A.); ghj163@kookmin.ac.kr (S.O.); tiennv@kookmin.ac.kr (T.V.N.)

**Keywords:** synapse-neuron-aware training, defect-tolerant neural networks, defective memristor crossbars, memristor defects, neuromorphic

## Abstract

To overcome the limitations of CMOS digital systems, emerging computing circuits such as memristor crossbars have been investigated as potential candidates for significantly increasing the speed and energy efficiency of next-generation computing systems, which are required for implementing future AI hardware. Unfortunately, manufacturing yield still remains a serious challenge in adopting memristor-based computing systems due to the limitations of immature fabrication technology. To compensate for malfunction of neural networks caused from the fabrication-related defects, a new crossbar training scheme combining the synapse-aware with the neuron-aware together is proposed in this paper, for optimizing the defect map size and the neural network’s performance simultaneously. In the proposed scheme, the memristor crossbar’s columns are divided into 3 groups, which are the severely-defective, moderately-defective, and normal columns, respectively. Here, each group is trained according to the trade-off relationship between the neural network’s performance and the hardware overhead of defect-tolerant training. As a result of this group-based training method combining the neuron-aware with the synapse-aware, in this paper, the new scheme can be successful in improving the network’s performance better than both the synapse-aware and the neuron-aware while minimizing its hardware burden. For example, when testing the defect percentage = 10% with MNIST dataset, the proposed scheme outperforms the synapse-aware and the neuron-aware by 3.8% and 3.4% for the number of crossbar’s columns trained for synapse defects = 10 and 138 among 310, respectively, while maintaining the smaller memory size than the synapse-aware. When the trained columns = 138, the normalized memory size of the synapse-neuron-aware scheme can be smaller by 3.1% than the synapse-aware.

## 1. Introduction

With the advent of ‘Big Data’, traditional CMOS-based computing platforms such as CPU, GPU, and FPGA are unable to match the demands of real-time data processing [1,2,3]. To address the limitations of CMOS digital systems, emerging computing circuits such as memristor crossbars have been investigated as potential candidates for significantly increasing the speed and energy efficiency of next-generation computing systems, which are required for implementing future AI hardware [4,5,6,7].

Unfortunately, manufacturing yield remains a serious challenge in adopting next-generation computing systems based on emerging devices due to the limitations of immature fabrication technology. Memristor crossbars, for example, have various fabrication-related issues, such as defects and variations, despite demonstrating superior computing performance and higher energy efficiency than standard CMOS computing systems. Defects in memristor crossbars are commonly referred to as stuck-at-faults, which occur when malfunctioning devices become caught in the High Resistance State (HRS) or the Low Resistance State (LRS) [8,9,10,11]. Even when programming pulses are delivered to memristor cells that become stuck at faults such as HRS or LRS, they are unable to modify their resistance states. Stuck-at-faults are quite common and can be found in the majority of memristor crossbars. For example, it was reported that approximately 10% and 4% of memristor cells were measured as stuck-at-LRS and stuck-at-HRS, respectively [12].

Let us now look at how malfunctioning memristor devices can impair the performance of neural networks based on emerging devices like memristor crossbars. Vector Matrix Multiplication (VMM) is a fundamental computing function used in neural network’s training and inference. The VMM process can be achieved in memristor crossbars, where vector and matrix multiplication is performed utilizing the memristor’s voltage-current relationship based on Ohm’s equation [5,13,14,15,16]. The most significant advantage of VMM performed on the memristor crossbar is that the VMM processing capabilities may be enhanced in parallel by adding more columns to the crossbar. Because of its expandable and parallel computing design, the memristor crossbar is well suited for diverse neural network applications processing ‘Big Data.’ Unfortunately, even a single memristor defect can have a large impact on the VMM calculation, because a fault like stuck-at-LRS can drastically raise the column current of the crossbar to which the damaged cell belongs [12]. The unintentional neuron activation caused by defective cells can drastically decrease the training and inference accuracy of neural network based on emerging devices [12,16,17,18,19,20].

Two types of defect-aware schemes can be considered to compensate for performance degradation caused by memristor defect cells such as stuck-at-faults. The first is the synapse-aware scheme, which trains memristor crossbars while taking into account the types and locations of synapse defects [20,21,22]. In this case, a defect map containing information on both defect types and locations should be used during the training of memristor crossbars. The neural network’s synapses and neurons are mapped on a real memristor crossbar while taking defect types and locations into account. The degradation of neural network’s performance can thus be minimized by defect-aware training of real memristor crossbars with defects [23,24,25,26].

The second is the neuron-aware scheme. If some neurons are connected to columns with a high number of faulty cells, they are referred to as ‘severely-defective columns’ in the neuron-aware scheme. During the training of memristor crossbars, the severely defective columns are not permitted to participate in neural network’s operations such as training and inference [22]. By doing so, the neural network can prevent the defective columns from affecting the neuron activation during the training and inference.

**Figure 1:**
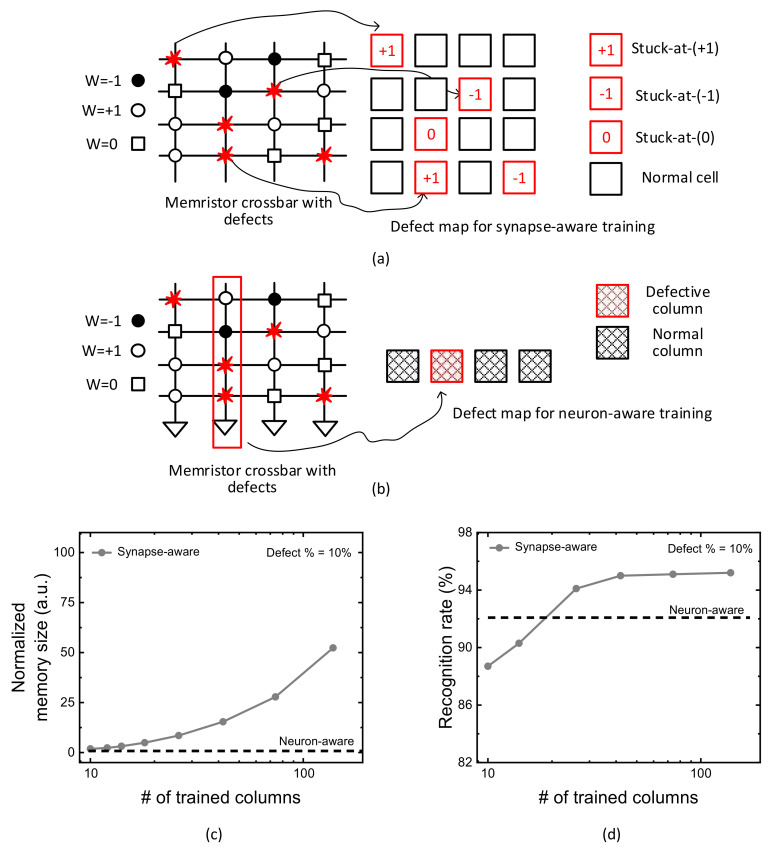
(**a**) The synapse-aware scheme for defective memristor crossbars (**b**) The neuron-aware scheme for defective memristor crossbars (**c**) Comparison of defect map’s memory size between the synapse-aware and neuron-aware schemes with increasing the number of crossbar’s columns trained for synapse defects, when the crossbar’s defect percentage = 10%. The total number of columns in the crossbar is 300 + 10. (**d**) Comparison of MNSIT recognition rate between the synapse-aware and neuron-aware with increasing the number of crossbar’s columns trained for synapse defects, when the crossbar’s defect percentage = 10%.

Figure 1a indicates a conceptual diagram of synapse-aware scheme, where a memristor crossbar with defects and its defect map are shown in left and right, respectively. Here, W means a synaptic weight. For ternary neural networks, the weight can be either −1, +1, or 0. W = −1, +1, and 0 are represented with solid circle, open circle, and open box, in Figure 1a, respectively [26]. In the crossbar, the defect cells are represented with red flames. The defect map for the synapse-aware scheme is also shown in right in Figure 1a. The red and black boxes represent faulty and normal cells, respectively. The fault types can be stuck-at-(+1), stuck-at-(−1), and stuck-at-(0), as indicated in Figure 1a. The defect location can be represented with the faulty cell’s row and column numbers stored in the defect map’s memory.

Figure 1b shows a conceptual diagram of the neuron-aware scheme. Like Figure 1a, a defective memristor crossbar and its map of defective columns are shown in left and right, respectively. In Figure 1b, the defect map contains only the information of defective columns. The red and black boxes represent the defective and normal columns, respectively. The defect map of the neuron-aware scheme requires much smaller memory than the synapse-aware, because only the defective column’s locations are stored in the defect map’s memory in Figure 1b.

Figure 1c compares the defect map’s memory size between the synapse-aware and neuron-aware schemes. In Figure 1c, the y-axis is the normalized memory size required for storing the defect maps of the synapse-aware and neuron-aware schemes. The memory size of defect map in the synapse-aware scheme can be calculated by multiplying the number of faulty cells by each defect’s memory size. The defect map’s memory size for considering faulty cells in the synapse-aware scheme includes both row and column numbers and defect types in the crossbar. On the contrary, for the neuron-aware, the defect map’s memory size can be obtained by multiplying the number of defective columns by each defective column’s memory size. Unlike the synapse-aware, the neuron-aware scheme uses only the information of defective column’s locations during the training.

In Figure 1c,d, the x-axis is the number of crossbar’s columns trained for considering faulty cells in the synapse-aware scheme. For the neuron-aware scheme, however, it should be noted that the number of deactivated columns during the neural network’s operation is fixed not changed in Figure 1c,d. For fixing the number of deactivated columns, we obtained an optimal number of crossbar’s columns that should be removed from the neural network’s operation from the simulation. The optimal number of deactivated columns obtained from the simulation can maximize the neural network’s performance trained with the neuron-aware scheme. In Figure 1c,d, the optimal number of crossbar’s columns deactivated during the training is 50, which was obtained from the simulation of the neuron-aware scheme. By doing so, the defect map’s memory size and the recognition rate of the neuron-aware scheme can be kept constant regardless of changing the number of crossbar’s columns trained for considering faulty cells in the synapse-aware scheme, as shown in Figure 1c,d.

Here, an artificial neural network used for calculating the memory size in Figure 1c,d is assumed to have 784 input, 300 hidden, and 10 output neurons for recognizing MNIST dataset [12,27]. For implementing the network, 784 × 300 and 300 × 10 crossbars are used for the VMM calculation. Here the total numbers of crossbar columns are 300 and 10 for the hidden and output neurons, respectively.

Figure 1c indicates very clearly that the neuron-aware scheme in Figure 1b needs much smaller defect map’s memory size than the synapse-aware in Figure 1a. As explained earlier, the neuron-aware scheme uses only the information of defective columns, while the synapse-aware needs the information of all the faulty cells during crossbar’s training. Because the number of faulty cells in the synapse-aware scheme outnumbers the number of the defective columns in the neuron-aware, the neuron-aware can train the defective crossbar using much smaller defect map’s memory size than the synapse-aware. Moreover, the defect map’s memory size of the neuron-aware scheme can be kept constant regardless of changing the number of crossbar’s columns trained for considering faulty cells in the synapse-aware scheme.

Figure 1d compares the recognition rate between the synapse-aware and neuron-aware schemes. In Figure 1d, the y-axis is MNIST recognition rate calculated by counting the number of successful and failed recognitions when the 10,000 MNIST test vectors are applied to the trained crossbar. The comparison in Figure 1d indicates that the neuron-aware scheme exceeds the synapse-aware in terms of recognition rate, when the number of crossbar’s columns trained for considering synaptic defects is small. In the neuron-aware scheme, the columns which are defined as ‘severely-defective’ are deactivated during the training and inference to prevent them from being involved in the neural network’s operation. By doing so, the neuron-aware can recognize MNIST images better than the synapse-aware, when the number of crossbar’s columns trained for considering synapse defects is small. However, as the number of crossbar’s columns trained with defects is increased, the synapse-aware begins to crossover the neuron-aware scheme, as indicated in Figure 1d. The crossover of recognition rate between the synapse-aware and neuron-aware is caused from the columns trained with defects can mitigate the degradation of neural network’s performance due to various stuck-at-faults in the crossbar.

In this paper, a synapse-neuron-aware training method is newly proposed, where the synapse-aware scheme is combined with the neuron-aware, for exploiting trade-off relationship between the defect map size and the neural network’s performance. In the synapse-neuron-aware training, the memristor crossbar’s columns are divided into 3 groups. The Group-1 columns that are defined as ‘severely defective’ are deactivated during the training and inference, like the neuron-aware scheme. The Group-2 columns that are defined as ‘moderately defective’ are trained by the synapse-aware scheme, where the defect map containing the synapse defect information such as types and locations is used for training the crossbar. The Group-3 columns containing less defects than Group-1 and Group-2 are defined as ‘normal columns’. For the Group-3, they are trained without considering defects. In the synapse-neuron-aware scheme proposed in this paper, each group is trained according to the trade-off relationship between the neural network’s performance and the hardware overhead of defect-tolerant training. As a result of this group-based training, the synapse-neuron-aware scheme can achieve the neural network’s performance better than the neuron-aware method with less hardware overhead than the synapse-aware one. By combining the synapse-aware with the neuron-aware together, in this paper, we can succeed in reducing the hardware burden of the synapse-aware method and improving the network’s performance better than the neuron-aware.

## 2. Method

**Figure 2:**
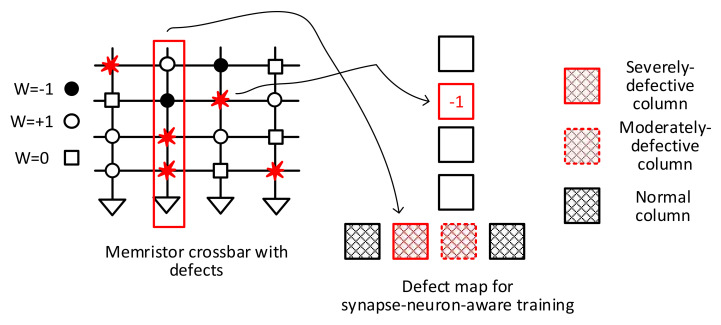
The synapse-neuron-aware scheme proposed for training memristor crossbars with defect cells.

A new synapse-neuron-aware scheme for considering faulty memristor cells is proposed in this paper. Figure 2 depicts a conceptual diagram of the proposed synapse-neuron-aware scheme, where the neuron-aware scheme is combined with the synapse-aware one to optimize both the neural network performance and the hardware overhead at the same time.

To begin using the synapse-neuron-aware training method, the crossbar’s columns are divided into three groups, as mentioned earlier. They are Group-1, Group-2, and Group-3, respectively. As shown in Figure 2, Group-1 represents the severely defective columns, which contain a large number of defect cells that should not be involved in the neural network’s operation. To do so, the Group-1 columns are deactivated during the training. They are unable to contribute to the neuron activation in the training and inference. The Group-2 columns defined as ‘moderately defective’ are trained with the synapse-aware scheme. The synapse defect map containing the synaptic defect information, such as types and locations, is used to train the Group-2 columns. The Group-3 columns with fewer defects than Group-1 and Group-2 are considered normal, and they are trained without taking defects into account during the training. For the Group-3 columns, no defect map is used.

Each group in the synapse-neuron-aware scheme is trained based on the trade-off relationship between the network performance and the hardware overhead of defect-tolerant training. As a result of this group-based training, the synapse-neuron-aware training method outperforms the neuron-aware method in terms of neural network performance while requiring less hardware overhead than the synapse-aware method. By combining the synapse-aware and neuron-aware methods together, the hardware burden of the synapse-aware method can be reduced and the network performance can be improved better than the neuron-aware method.

**Figure 3:**
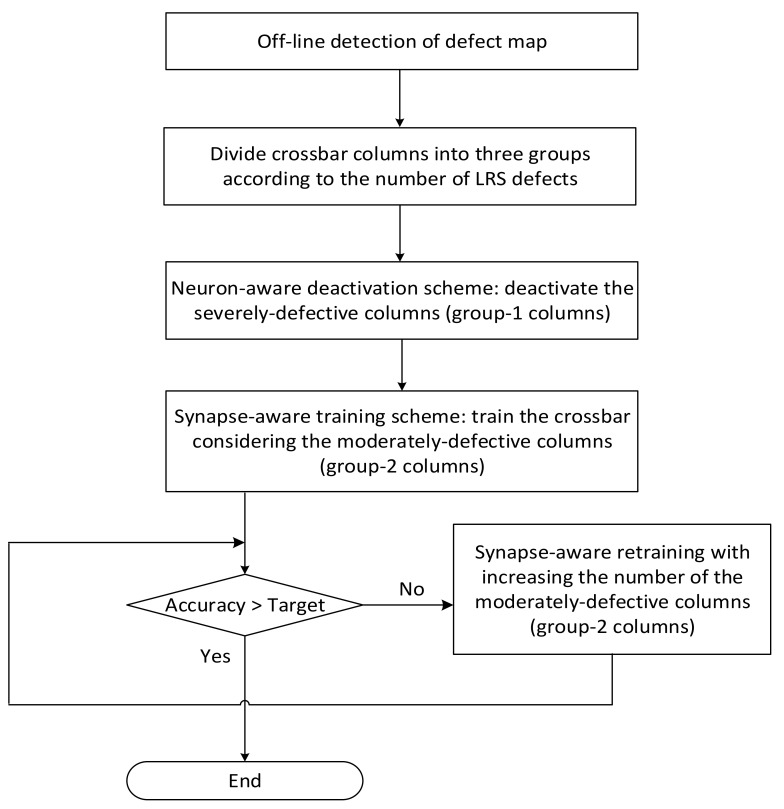
The flowchart of synapse-neuron-aware training scheme.

**Figure 4:**
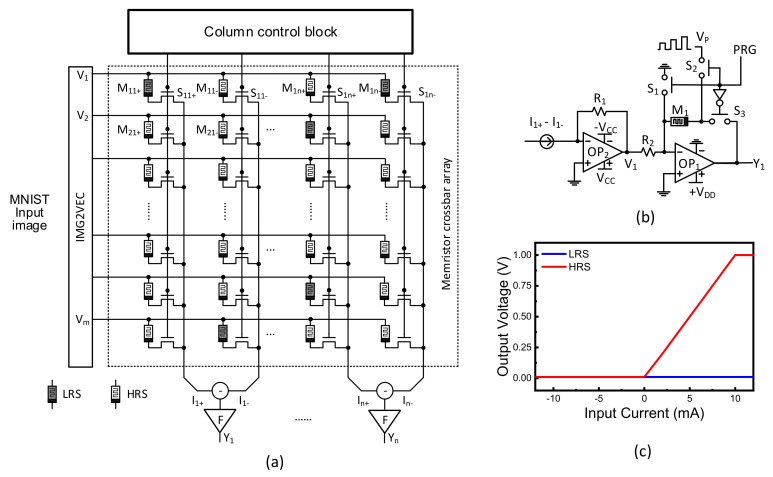
(**a**) The schematic of a 1T-1M crossbar composed of memristors and transistors [28,29,30] (**b**) The ReLU activation function circuit with column deactivation (**c**) The transfer curve for ReLU activation function with column deactivation. The red line is for the ReLU transfer curve. The blue line is for the column deactivation.

Figure 3 shows a flowchart of the proposed synapse-neuron-aware scheme for training memristor crossbars with defects. In Figure 3, first, a defect map is obtained from the crossbar measurement. Using the measured defect map, the crossbar columns can be divided into three groups of Group-1, Group-2, and Group-3. The Group-1 has the severely-defective columns and they should be deactivated to keep them from being involved in the neural network operation. If the Group-1 columns are involved in the neural network operation, they may be activated very frequently regardless of the input vectors. The frequent activation of severely-defective columns can degrade the neural network performance significantly. To avoid the unwanted neuron activation, the severely-defective columns are deactivated by controlling the activation function circuit as will be explained in Figure 4. After deactivating the severely-defective columns (the Group-1 columns), the memristor crossbar is trained considering the synapse defects belonging to the moderately-defective columns (the Group-2 columns), as indicated in Figure 3. After training the memristor crossbar with defects, the inference accuracy is tested. If the accuracy does not meet a target rate, the crossbar is trained again with increasing the number of Group-2 columns until the target accuracy is satisfied.

Figure 4a shows a memristor crossbar composed of transistors and memristors. Here V_1_, V_2_, etc. represent input voltages. To calculate both positive and negative synaptic weights, two columns are used in the crossbar, which are denoted with (+) and (−) symbols in Figure 4a. I_1+_ and I_1−_ represent positive and negative column currents, respectively. I_1_ is calculated from the difference of I_1+_ and I_1−_. M_11+_ and M_11−_ are memristors for positive and negative columns, respectively. S_11_ is a selector for controlling the access to M_11+_ and M_11−_. In Figure 4a, F means the activation function circuit, which is shown in detail in Figure 4b. Y_1_, Y_2_, etc. represent output voltages from output neurons. Here ternary synaptic weights are used in Figure 4a. For the synaptic weight = 0, both memristors on positive and negative columns are HRS. For the weight = 1, the positive and negative cells are LRS and HRS, respectively. If the positive and negative columns have HRS and LRS, the weight can be −1, respectively.

Figure 4b shows a ReLU activation function circuits with column deactivation. In the activation circuit, OP_2,_ acts as a current-to-voltage converter. OP_1_ is an inverting amplifier. If a column has many memristor defects, this column can be defined as ‘severely-defective column (Group-1)’. The column belonging to the Group-1 should be deactivated during the training time. By doing so, the column is not involved in the neural network operation. For deactivating the column, M_1_ is programmed with LRS in Figure 4b. By doing so, the OP1’s amplifying gain becomes very small. S_1_, S_2_, and S_3_ are controlling switches. V_P_ means a memristor programming pulse, which is applied to M_1_ through S_1_ and S_2_. S_1_ and S_2_ are turned on when PRG is high. On the contrary, when PRG is low, S_3_ is turned on. In this case, the column current of I_1+_-I_1−_ is converted to the node voltage of V_1_, according to R_1_. Figure 4c shows the column current of I_1+_-I_1_ is converted to the output voltage of Y_1_, when M_1_’s conductance is 1/HRS and 1/LRS, respectively. The blue line means the column is deactivated. The red line is for the ReLU transfer curve.

## 3. Results

**Figure 5:**
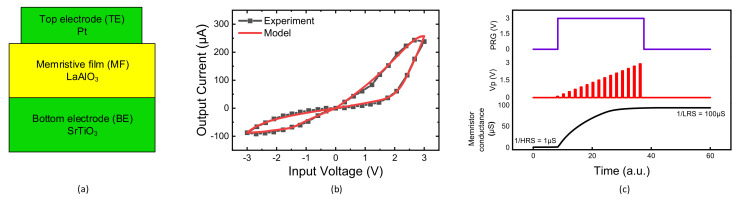
(**a**) The cross-sectional view of memristor used in this paper [31,32]. (**b**) The memristor’s butterfly curve (**c**) The memristor’s programming transient characteristic used in the ReLU activation function circuit with column deactivation.

Figure 5a shows a cross-sectional view of the memristor that was used in this paper. The measured memristor has a film structure of Pt/LaAlO_3_/Nb-doped SrTiO_3_ stacked layer [31,32]. Here, the top electrode layer (TE) is formed with Platinum (Pt). The bottom electrode (BE) is made of SrTiO3. The TE size is 100 µm × 100 µm. The detailed fabrication process is explained in the previous publication [32]. Figure 5b shows a memristor’s butterfly curve, where HRS and LRS are 1 MΩ and 10 kΩ, respectively [31]. Here the symbol and line represent the measurement and the Verilog-A simulation, respectively. The mathematical model equations describing the measured current-voltage relationship can be found in the previous publication [31]. The equations were implemented in Verilog-A in the CADENCE SPECTRE (Cadence Design Systems, Inc., San Jose, CA, USA), which is used for the hybrid circuit simulation of memristors and complementary metal-oxide-semiconductor (CMOS) circuits in this paper. The measurement was performed with Keithley-4200 (Semiconductor Characterization System, Tektronix, Inc., Beaverton, OR, USA) combined with the Keithley-3706 Switching Matrix.

Figure 5c shows a memristor’s programming transient characteristic with applying the programming voltage pulses with amplitude modulation, as shown in Figure 4b. Initially, the memristor in Figure 4b is programmed with HRS [25]. As the voltage pulses are applied, the memristor’s conductance is increased from 1/HRS to 1/LRS. The smallest conductance of memristor can deactivate the corresponding column during the training and inference, because the amplifier gain becomes very small. By doing so, the corresponding crossbar’s column can be ignored in the neuron activation.

**Figure 6:**
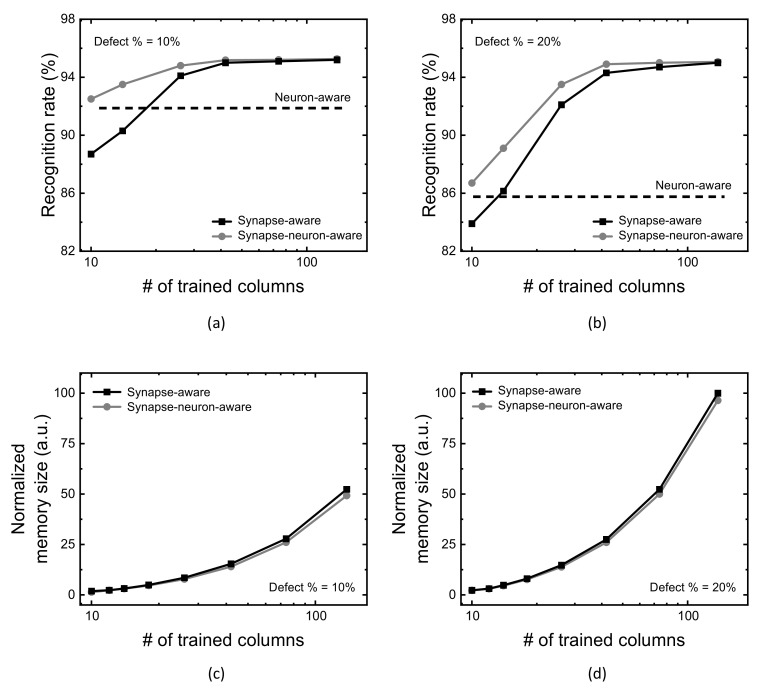
(**a**) MNSIT recognition rate with increasing the number of crossbar’s columns trained for synapse defects (Group-2 columns), when the crossbar’s defect percentage = 10%. (**b**) MNIST recognition rate with increasing the number of crossbar’s columns trained for synapse defects (Group-2 columns), when the crossbar’s defect percentage = 20%. (**c**) Defect map’s memory size for the crossbar’s defect percentage = 10% (**d**) Defect map’s memory size for the crossbar’s defect percentage = 20%.

Figure 6a compares MNIST recognition rate of the neuron-aware, synapse-aware, and synapse-neuron-aware scheme, with changing the number of crossbar’s columns trained for synapse defects (Group-2 columns). Here the crossbar’s defect percentage is assumed 10% in Figure 6a. The assumption of defect percentage = 10% comes from the measured defect percentage of stuck-at-faults was as much as 10% in the fabricated memristor crossbars [12,33]. For the neuron-aware scheme, first, the severely-defective columns are deactivated in order to keep them from being involved in the neural network’s operation. In Figure 6a, the severely-defective 50 columns are deactivated from the neuron activation among 300 and 10 columns of the hidden neurons and output neurons, respectively. Because of the deactivation, when the number of crossbar’s columns trained for synapse defects is very small, the neuron-aware scheme can show the rate better than that of the synapse-aware, in Figure 6a.

On the contrary, as the number of crossbar’s columns trained for synapse defects is increased, the synapse-aware scheme beings to exceed the neuron-aware. As explained earlier, the neuron-aware scheme does not consider the synapse defects during the training. Hence, the recognition rate of the neuron-aware scheme is constant regardless of increasing the number of crossbar’s columns trained for synapse defects, while the synapse-aware scheme can improve the rate.

The synapse-neuron-aware scheme was proposed as the combination of the synapse-aware and neuron-aware, explained in the previous Section 2. As a result of the combination of two schemes, the synapse-neuron-aware scheme acts similarly with the neuron-aware, when the number of crossbar’s columns trained for synapse defects is small. As the number of crossbar’s columns trained for synapse defects is increased, the combined scheme seems to follow the synapse-aware, not acting like the neuron-aware. In Figure 6a, with increasing the number of crossbar’s columns trained from 10 to 138, the synapse-neuron-aware scheme shows the recognition rate better than the both the synapse-aware and neuron-aware in the entire range of trained columns.

Similarly with Figure 6a,b compares the recognition rate of the neuron-aware, synapse-aware, and synapse-neuron-aware scheme, with changing the number of crossbar’s columns trained for synapse defects (Group-2 columns), for the crossbar’s defect percentage = 20%.

Figure 6c,d compare defect map’s memory size between the synapse-aware and synapse-neuron-aware schemes. The comparison in these figures indicates the synapse-neuron-aware requires a little smaller memory size of defect map than the synapse-aware. This is because the memory size of defect map of the neuron-aware is negligibly small. In addition, the crossbar’s column deactivation of the severely-defective columns can reduce the memory size of synapse-defect map in the synapse-neuron-aware scheme. This is due to the fact that the synapse-neuron aware scheme does not store the information of faulty cells belonging to the severely-defective columns that are removed from the neuronal network’s operation. On the contrary, the synapse-aware scheme should store the information of all the faulty cells in the defect map, which requires more memory size than the synapse-neuron aware.

The proposed training method was also tested for CIFAR-10 dataset, which contains RGB colors images. Here, the conventional ResNet architecture is used for simulating the proposed synapse-neuron-aware scheme with CIFAR-10 dataset [34,35]. Like the neural network architecture for testing the MNIST dataset, 300 hidden neurons and 10 output neurons are used for implementing the fully-connected layers in ResNet architecture [36,37].

**Figure 7:**
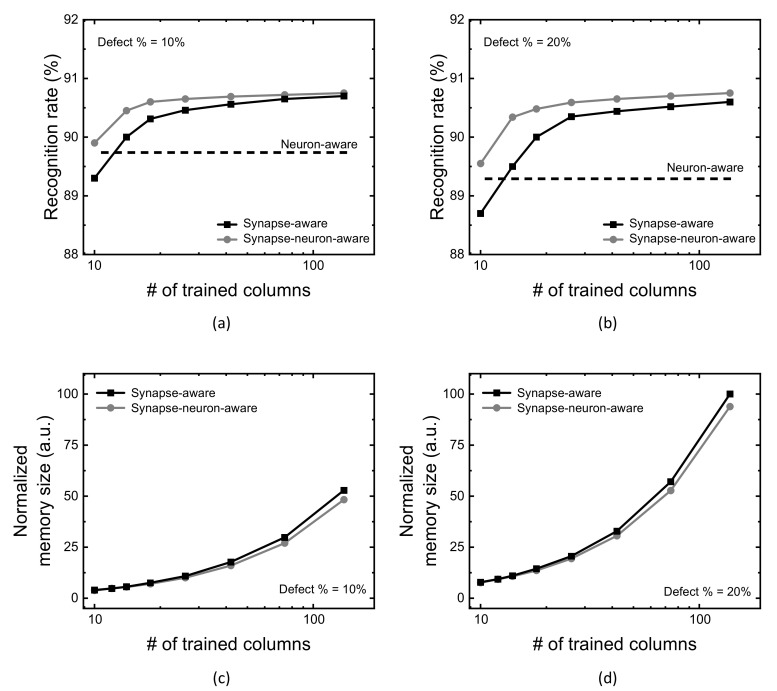
(**a**) CIFAR-10 recognition rate with increasing the number of crossbar’s columns trained for synapse defects (Group-2 columns), when the crossbar’s defect percentage = 10%. (**b**) CIFAR-10 recognition rate with increasing the number of crossbar’s columns trained for synapse defects (Group-2 columns), when the crossbar’s defect percentage = 20%. (**c**) Defect map’s memory size for the crossbar’s defect percentage = 10% (**d**) Defect map’s memory size for the crossbar’s defect percentage = 20%.

Figure 7a,b compare CIFAR-10 recognition rate of the neuron-aware, synapse-aware, and synapse-neuron-aware scheme, for the crossbar’s defect percentage = 10% and 20%, respectively. In Figure 7a, when the number of crossbar’s columns trained for synapse defects is 10, the gap between the synapse-neuron-aware and the synapse-aware is 0.6% for ResNet architecture. To verify the proposed scheme useful for various neural networks, VGG16 architecture is also tested in this paper [38]. From the VGG16 simulation, the synapse-neuron-aware indicates CIFAR-10 recognition rate better by 0.8% than the synapse-aware, which is very similar with the ResNet simulation result, when the number of crossbar’s columns trained for synapse defects is 10. Figure 7c,d compare the memory size between the synapse-neuron-aware and synapse-aware, for the crossbar’s defect percentage = 10% and 20%, respectively.

Considering both the recognition rate and memory size in Figure 7a,c, respectively, for the defect percentage = 10%, the trade-off relationship between the neural network’s performance and hardware overhead is indicated clearly. As the number of trained columns for considering synapse defects is increased, the recognition rate becomes improved. In contrast, the memory overhead due to the defect map is degraded. Reversely, if the number of columns trained in the crossbar is decreased, the recognition rate becomes degraded, while the defect map’s size becomes small. Though the synapse-aware and the proposed synapse-neuron-aware show the same trade-off relationship between the neural network’s performance and hardware overhead, there is one thing different between the proposed and the synapse-aware schemes, as indicated in Figure 7a. In the entire range of the number of trained columns, the proposed scheme can show better recognition rate than the synapse-aware, as indicated in Figure 7a,b, because the defective column deactivation compensates for the performance degradation caused from severely-faulty columns.

Finally, one thing to note here is that the synapse-neuron-aware scheme proposed in this paper can be more suitable to the on-line training applications compared to the off-line software training. This is owing to the fact that the proposed scheme can demonstrate the neural network’s performance better than the synapse-aware, in spite of using a smaller memory size of defect map. Especially, for the edge intelligence applications, where the embedded memory size can be very limited, the smaller defect map’s memory size of the proposed scheme can alleviate a hardware burden of the on-device training significantly.

## 4. Conclusions

To overcome the limitations of CMOS digital systems, emerging computing circuits such as memristor crossbars have been investigated as potential candidates for significantly increasing the speed and energy efficiency of next-generation computing systems, which are required for implementing future AI hardware. Unfortunately, manufacturing yield still remains a serious challenge in adopting memristor-based computing systems due to the limitations of immature fabrication technology.

To compensate for the malfunction of neural networks caused from the fabrication-related defects, the new crossbar training scheme combining the synapse-aware with the neuron-aware together was proposed for exploiting trade-off relationship between the defect map size and the neural network’s performance, in this paper. In the proposed scheme, the memristor crossbar’s columns are divided into the 3 groups, which are the severely-defective, moderately-defective, and normal columns, respectively, according to the number of LRS defects per columns. Here, each group can be trained according to the trade-off relationship between the neural network’s performance and the hardware overhead of defect-tolerant training.

As a result of this group-based training, the synapse-neuron-aware scheme could achieve the neural network’s performance better than the neuron-aware method while using less hardware overhead than the synapse-aware one. For example, when testing the defect percentage = 10% with MNIST dataset, the proposed scheme outperformed the synapse-aware and the neuron-aware by 3.8% and 3.4% for the number of crossbar’s columns trained for synapse defects = 10 and 138 among 310, respectively, while maintaining the smaller memory size than the synapse-aware. When the trained columns = 138, the normalized memory size of the synapse-neuron-aware scheme could be smaller by 3.1% than the synapse-aware.
